# AveloMask, a novel breath aerosol collection kit for airborne *Mycobacterium tuberculosis*: a proof-of-principle assessment

**DOI:** 10.1128/jcm.00546-25

**Published:** 2025-08-21

**Authors:** Patricia Risch, Tobias Broger, Zandile Booi, Katie Tiseo, Harshitha Santhosh Kumar, Jamie van Schalkwyk, Theresa Heinrich, Reto Willi, Stefan M. Botha, Peter Sander, Adithya Cattamanchi, Stephan Hubold, Claudia M. Denkinger, Grant Theron, Christina Fialová, Christian Adlhart, Rouxjeane Venter

**Affiliations:** 1 Avelo AGSchlieren, Switzerland; 2 DSI-NRF Centre of Excellence for Biomedical Tuberculosis Research, South African Medical Research Council Centre for Tuberculosis Research, Division of Molecular Biology and Human Genetics, Faculty of Medicine and Health Sciences, Stellenbosch University 121470, Cape Town, South Africa; 3 Institute of Medical Microbiology, University of Zurich 27217, Zurich, Switzerland; 4 BLINK AGJena, Germany; 5 National Reference Laboratory for MycobacteriaZurich, Switzerland; 6 Center for Tuberculosis, University of California San Francisco 8785, San Francisco, California, USA; 7 Department of Medicine, Division of Pulmonary Diseases and Critical Care Medicine, University of California Irvine 8788, Irvine, California, USA; 8 Department of Infectious Diseases and Tropical Medicine, Heidelberg University Hospital 64338 , Heidelberg, Germany; 9 German Center for Infection Research (DZIF), partner site Heidelberg University Hospital 27178, Heidelberg, Germany; 10 ZHAW Zurich University of Applied Sciences, School of Life Sciences and Facility Management, Institute of Chemistry and Biotechnology 117457 Wädenswil, Switzerland; University of Manitoba, Winnipeg, Manitoba, Canada

**Keywords:** tuberculosis, diagnosis, non-sputum, aerosol, breath, face mask sampling, accuracy, PCR

## Abstract

Tuberculosis (TB) remains the world’s deadliest infectious disease, with many active cases missed due to challenges in sputum collection. Exhaled breath aerosols (XBA), a major route of *Mycobacterium tuberculosis* (MTB) transmission, offer a promising non-invasive alternative. This study evaluated the diagnostic accuracy and feasibility of the AveloMask—a novel point-of-care breath aerosol collection kit—for detecting active pulmonary TB using quantitative PCR (qPCR). In a pilot diagnostic accuracy study, 61 symptomatic, adult outpatients in Cape Town, South Africa, wore the mask for 45 min, coughing deeply at the start and end. XBAs were collected on integrated fiber filters transferred into stabilizing buffer via a simple push step and biobanked. XBA’s were batch-analyzed by qPCR targeting the MTB-specific *IS6110* sequence. Diagnostic accuracy was assessed against sputum Xpert MTB/RIF Ultra (SXRS) and a composite microbiological reference standard (MRS), including culture. Of the 58 evaluable participants, 59% (34/58) had confirmed TB. Compared with SXRS, mask qPCR showed 71.0% (95% confidence interval [CI]: 53.4%–83.9%) sensitivity and 92.3% (95% CI:75.9%–97.9%) specificity. Against MRS, sensitivity was 64.7% (95% CI: 47.9%–78.5%) and specificity 91.7% (95% CI: 74.2%–97.7%). Sensitivity increased with bacterial load, reaching 100% in sputum with high MTB concentrations. MTB *IS6110* copy numbers in XBAs were low overall (175 copies [4–2,147]), likely due to insufficient DNA recovery or low aerosol bacilli. The mask sampling was well-tolerated by users. The AveloMask Kit shows promising diagnostic accuracy for TB and is feasible for point-of-care use. Further optimization and larger validation studies are warranted.

**IMPORTANCE:**

Tuberculosis (TB) remains the world’s deadliest infectious disease, yet diagnosis still relies heavily on sputum, which many patients struggle to produce. This study introduces the AveloMask Kit, a user-friendly, non-invasive face mask that captures exhaled aerosols and transfers them into a buffer tube for molecular detection of respiratory tract infections. In a clinical proof-of-principle study, AveloMask detected TB with promising accuracy and demonstrated feasibility in outpatient settings. By offering a non-invasive alternative to sputum, the AveloMask Kit addresses a critical diagnostic gap and could expand access to TB testing, particularly in resource-limited or primary care settings. Its simplicity enables use by minimally trained staff, and its stabilizing buffer allows ambient-temperature transport and biobanking, supporting broader case finding, safer sample collection, and future aerobiology research.

## INTRODUCTION

Worldwide, tuberculosis (TB) reclaimed its position as the deadliest infectious disease in 2023, claiming 1.25 million lives that year (1). If Sustainable Development Goal (SDG) targets are not met, TB is projected to cause 31.8 million deaths and economic losses of US$17.5 trillion over the next 30 years (2). Currently, 25% of TB cases (2.7/10.8 million) go undiagnosed (1). A major contributor to this diagnostic gap is that 7.6% to 18% of individuals with TB are unable to produce sputum, the primary specimen used for bacteriological detection of *Mycobacterium tuberculosis* (MTB) (3–5). Testing on alternative non-sputum-based samples has been identified as an important priority to increase diagnostic yield and help close the diagnostic gap (6, 7).

Since antiquity, it has been known that breath contains clues to many diseases (8). Breath is an attractive sample type due to its non-invasive and simple collection process. In recent years, breath-based diagnostic research has primarily focused on volatile organic compounds (VOCs), which originate from metabolic processes. However, VOC-based diagnostics often lack specificity, as VOC profiles can be influenced by multiple diseases, physiological conditions, and host factors. A recent meta-analysis found a pooled specificity of 83% for VOC-based TB detection, with high heterogeneity (9).

An alternative approach is the collection and testing of exhaled breath aerosols (XBA). Aerosols are microscopic liquid and solid particles with a size between approx. 10 nm and 100 µm that can carry pathogens, nucleic acids, and proteins (10). Aerosol collection and testing has the potential for higher specificity than VOC-based testing, as it enables direct detection of pathogen markers, such as nucleic acids, with molecular tests. Moreover, because breath aerosols are linked to respiratory pathogen transmission, XBA-based detection could help identify individuals most likely to spread infection (11). Growing evidence supports aerosol transmission as a major route for many respiratory infections, including pandemic coronaviruses, influenza, respiratory syncytial virus (RSV), and TB (10, 12–16). Thus far, aerosol collection for pathogen detection was primarily done in academic research laboratories using techniques, such as impingers, impactors, or precipitators, which require relatively high technical efforts (12, 17–19). For the diagnosis of active TB, others have used face mask sampling (FMS) with PCR detection and have reported sensitivities ranging from 65% to 86% (20–23). An important operational consideration for FMS is the removal and processing of the aerosol capture filter for subsequent testing, which is typically done aseptically with forceps, prone to contamination, and often impractical at the point of collection. Other key aspects are the highly efficient aerosol capture and release combined with compatibility of the recovered aerosol sample with downstream extraction, molecular testing, and sample transport stability. To address these challenges, we have developed an easy-to-use FMS kit (AveloMask Kit, Avelo AG, Switzerland) for the collection, concentration, transport, and storage of human breath aerosol specimens.

In this study, we aimed to assess the feasibility and diagnostic accuracy of AveloMask collection combined with quantitative polymerase chain reaction (qPCR) for the detection of active TB in adult outpatients, compared against a composite microbiological reference standard (MRS) and sputum Xpert MTB/RIF Ultra (Xpert Ultra; Cepheid). In addition, we aimed to quantify MTB *IS6110* copies recovered from mask samples and to evaluate losses due to incomplete lysis to inform further protocol improvements.

## MATERIALS AND METHODS

### AveloMask kit design and procedure

**Fig 1:**
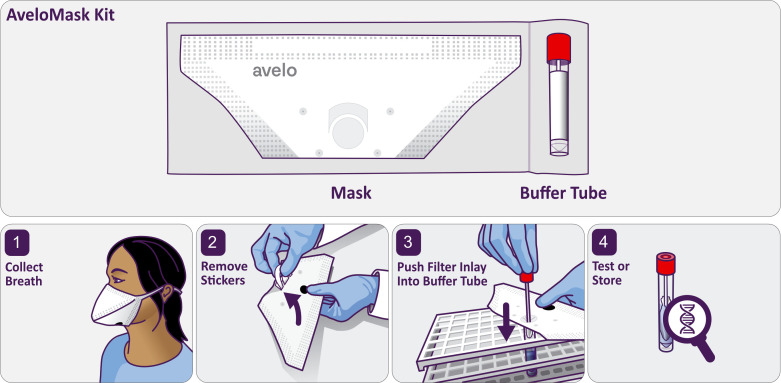
AveloMask Kit and breath aerosol collection procedure.

AveloMask (Avelo AG, Switzerland) is a specimen collection kit for the collection of a person’s exhaled breath aerosol (XBA) sample from the respiratory tract. The processing steps are illustrated in Fig. 1. The kit contains a mask and a buffer tube. The inner side of the mask contains a filter inlay to collect aerosol particles from the exhalate. The filter inlay is a 75 × 75 mm sheet with electro-spun fibers mounted to the inside of the mask (Supplemental methods). After wearing the mask (step 1), two stickers from each side of the mask, which protect the inlay during wear, are peeled off (step 2). The filter inlay is removed by pushing it into the buffer tube using a stick attached to the tube cap (step 3). The buffer inactivates the sample and preserves nucleic acids for transport of the sample at ambient temperature until further processing or biobanking (step 4). Detailed instructions are given in Fig. S1 and Movie S1.

### Analytical evaluation

The aerosol filtration efficiency of the filter inlay was evaluated according to EN13274-7 using a PMFT 1000 test system (Palas, Germany). The assessment was conducted on 10 representative filter areas of 56 cm² with paraffin oil aerosol particles generated by a PLG 1000 aerosol generator (Palas, Germany). Aerosol size distribution was measured using a Promo 1000 aerosol photometer (Palas, Germany).

The capture of Mycobacterial aerosols on the filter inlay was tested by exposing the filter to a nebulized bacterial suspension of *Mycobacterium bovis* BCG tagged with green fluorescent protein (BCG-GFP 24; Supplemental methods).

### Clinical evaluation

#### Study participants

To demonstrate the efficient capture and detection of MTB in XBAs, we enrolled 61 participants between May 5 and October 17, 2024. We enrolled consenting adults (aged ≥18 years) with symptoms of TB at four outpatient primary health facilities in Kraaifontein, Scottsdene, Bloekombos, and Wallacedene (Cape Town, South Africa). Eligible participants had a persistent cough for at least 2 weeks and at least one additional symptom, such as hemoptysis, weight loss, fever, night sweats, malaise, contact with an active TB patient, chest pain, or loss of appetite. We excluded individuals currently receiving antimycobacterial treatment, those treated for TB in the past twelve months, and those unwilling to provide informed consent. In the initial phase, we enriched the study population by preferentially enrolling individuals with positive sputum Xpert Ultra results (*n* = 20). Thereafter, we enrolled participants consecutively (independent from the Xpert Ultra results) from the same population.

#### Ethics and reporting

The Stellenbosch University Health Research Ethics Committee (HREC) approved the study (No. N16/07/089). Written informed consent was obtained from patients, as per the study protocols. Study participation did not affect the standard of care. This study is reported in accordance with the Standards for Reporting of Diagnostic Accuracy Studies guidelines (see STARD checklist in Supplemental material) (25).

#### Sample collection and participant feedback

We collected detailed demographic and clinical data via standardized electronic case report forms (eCRFs) in a secure GCP/21 CFR part 11-compliant REDCap database (26). Sputum samples were obtained for reference standard testing with Xpert Ultra (Cepheid, USA) and MGIT culture after decontamination with 1% NaOH-NALC (Becton Dickinson, USA). The average interval between sputum collection and mask breath sampling was 1.8 days (range: 0–15 days). Participants wore the mask for 45 min, coughing deeply five times at the beginning and five times at the end of the collection period, in addition to any naturally occurring coughs. This sampling protocol was defined based on insights from the literature, particularly previous studies using 60 min collection periods (20), but reduced to 45 min due to operational feasibility in outpatient settings. The inclusion of coughing was informed by evidence that cough events release higher concentrations of MTB compared to tidal breathing alone (27).

To gather basic feedback on ease of use, participants were asked immediately after sample collection: “Overall, how difficult or easy was it to provide the breath sample? Very easy, somewhat easy, okay, somewhat difficult, very difficult.” Responses were recorded in the eCRFs.

Mask samples were processed on-site by trained community health workers. Filter inlays were transported in buffer at ambient temperature on the same day to the Stellenbosch University Biomedical Research Institute. There, they were stored at −20°C before being shipped on dry ice to Avelo, Switzerland, for blinded batch testing.

#### Mask sample processing and PCR analysis

The frozen mask samples (consisting of filter inlays in stabilizing guanidinium thiocyanate buffer) were thawed, vortexed for 30 s, and placed in a water bath at 95°C for 1 h. After the heat elution/lysis, a disposable, sterile Pasteur pipette was used to compress the filter inlay and transfer the liquid into a new tube for subsequent extraction. Then, 1.5 mL of molecular-grade ethanol was added, and DNA was extracted from the entire sample using the QIAamp DNA Mini Kit (Qiagen, Germany), following the manufacturer’s instructions—omitting proteinase K and buffer AL, as the sample buffer already contains guanidinium thiocyanate. The samples were extracted and eluted with 100 µL Buffer AE. Every batch included a negative and positive process control (buffer and buffer spiked with 50 colony-forming units BCG, respectively) and a five-level standard curve (example in Fig. S2). Four 9-µL aliquots of DNA extract per sample were tested in four separate reactions. qPCR was performed using a QuantStudio5 96-well 0.2-mL thermal cycler (Thermo Fisher, Switzerland) with well-established primers and probes (28) (Supplemental methods). To assess the completeness of lysis, a subset of filter inlays from TB-positive participants underwent a second extraction. This was accomplished by adding 3 mL of guanidinium thiocyanate buffer and repeating the same processing and extraction protocol as described above. Mask assessors were blinded to clinical information and reference standard results.

#### Digital PCR testing

After unblinding, leftover DNA extracts from two false-positive samples and a subset of true-positive and true-negative samples were retested using a novel digital PCR (dPCR) assay developed by BLINK to further investigate the presence of amplifiable MTB DNA and estimate copy numbers (Supplemental methods). The BLINK dPCR assay employs magnetic nanoreactor beads (mNRBs) loaded with the same primers and probes like the qPCR assay and is run on the BLINK X instrument (BLINK AG, Jena, Germany) (29). The assay enables absolute quantification of DNA copy number and includes melt curve analysis to verify the presence and size of the target amplicon.

### Reference standard definitions

We used two reference standards: a composite microbiological reference standard (MRS) and a sputum Xpert MTB/RIF Ultra-based reference standard (SXRS). Under the MRS, participants were classified as having active TB if they had at least one positive sputum Xpert Ultra result and/or at least one positive sputum culture. Participants were considered MRS-negative if all sputum cultures and Xpert Ultra tests were negative, requiring a minimum of one negative sputum culture result. For the SXRS, participants were considered to have active TB if they had positive sputum Xpert Ultra results (very low or higher semiquantitative categories). Participants with a sputum Xpert Ultra trace-positive results were classified as indeterminate for the SXRS but considered positive in the MRS. For false-positive mask results, participant records were reviewed in detail to extract information on chest X-ray, sputum smear microscopy, oral swab molecular results, antimycobacterial treatment, follow-up, and TB history, if available. Assessors of reference standard samples were blinded to index test results and clinical information.

### Data analysis

A precision-based approach was used to determine the sample size, targeting two-sided 90% Wilson-score confidence intervals with a width of ±20%, assuming 65% sensitivity for mask qPCR. The assumed sensitivity, being lower than specificity and the study’s primary focus, guided the calculation, requiring 20 TB-positive participants (30). At least an equal number of TB-negative participants was enrolled.

We calculated the point estimates and 95% Wilson confidence intervals for the sensitivity and specificity of mask qPCR by comparison with the MRS and SXRS. Mean *IS6110* copy numbers from the four qPCR replicates of mask samples were used for ROC curve analysis by comparison with the SXRS using the pROC R package. In an exploratory subgroup analysis, sensitivity and mean *IS6110* copy numbers were assessed across sputum Xpert Ultra semiquantitative categories, MGIT culture time to positivity (TTP), and sex, to explore potential performance differences related to the typically smaller exhaled breath volume in female than in male participants. To assess the completeness of lysis, we determined the percent recovery of *IS6110* copy numbers from the first and second extractions of the same filter inlay, with the combined sum of copies from both extractions set to 100%. Analyses were performed via GraphPad Prism (version 10) and R (version 4.4.2).

## RESULTS

### Analytical evaluation

**Fig 2:**
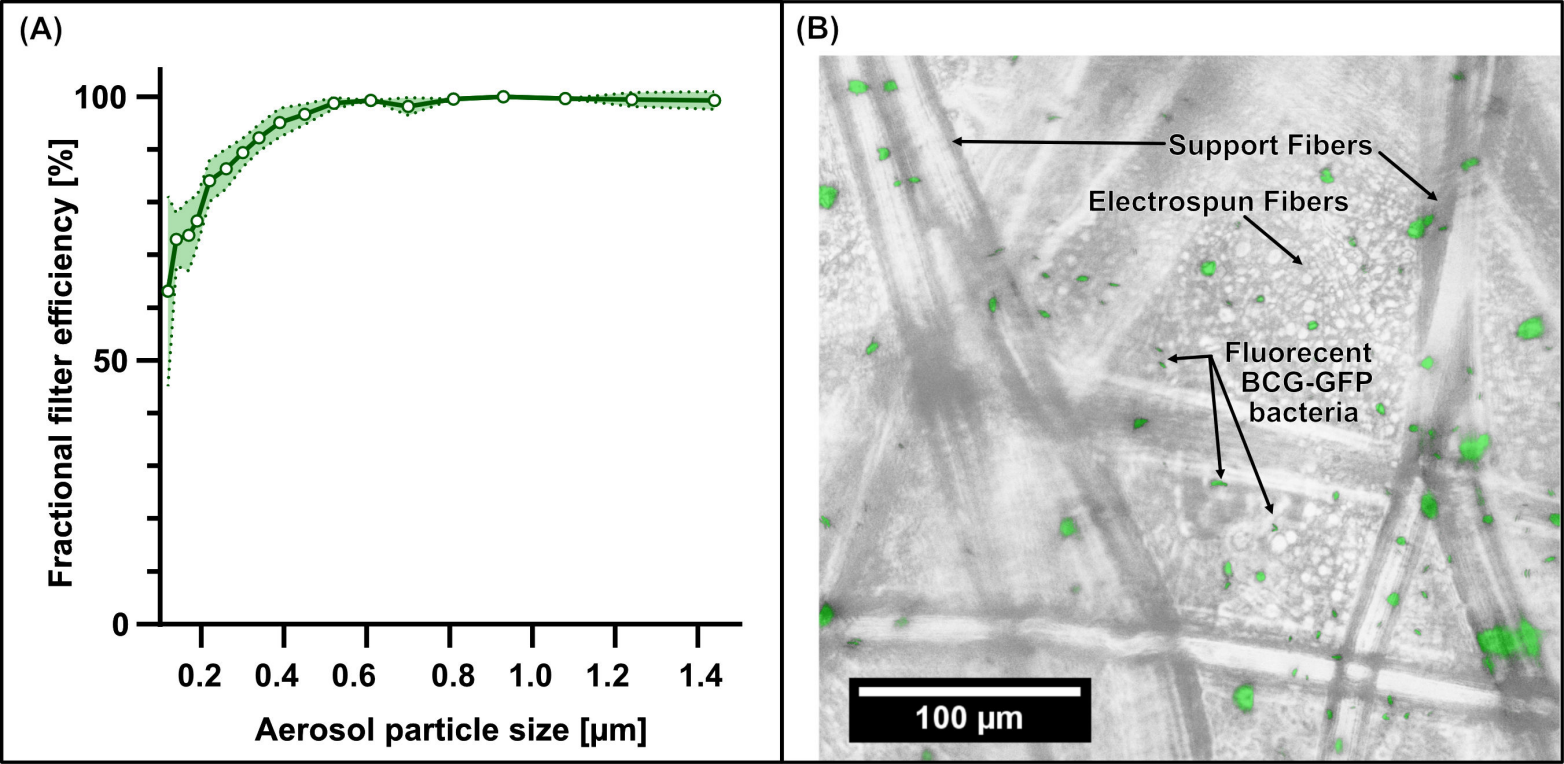
Filter inlay aerosol capture. (**A**) Fractional filtration efficiency (solid line) and 95% confidence intervals (dotted line) from 10 replicate tests of filter inlays showing high filtration efficiency for aerosol particle sizes above 0.3 µm, which is relevant for virus and bacteria. (**B**) Visualization of *Mycobacterium bovis* BCG tagged with green fluorescent protein (BCG-GFP) captured on the filter inlay. Green fluorescence channel image overlaid with a brightfield microscopy image showing rod-shaped BCG-GFP bacteria, indicating successful capture of nebulized mycobacterial aerosol.

Figure 2A shows the average fractional filtration efficiency of 10 filter inlays. The aerosol filtration efficiency of the filter inlay was greater than 80% for particles larger than 0.3 µm and efficiency was greater than 95% for particles larger than 0.5 µm (Table S1). The cumulative filtration efficiency of 95.8% (95% confidence interval [CI]: 94.5%–97.1%) for the particle size range of 0.3 to 2.2 µm suggests excellent capture of viral and bacterial aerosols by the filter inlay. Successful capture of bacterial aerosols was confirmed with nebulized BCG-GFP using microscopy (Fig. 2B). The compatibility of the filter material and buffer with nucleic acid extraction and downstream PCR was confirmed by spiking BCG onto the filter inlay and sample processing as described below, with no evidence of PCR inhibition observed.

### Clinical evaluation

#### Participant characteristics

**Fig 3:**
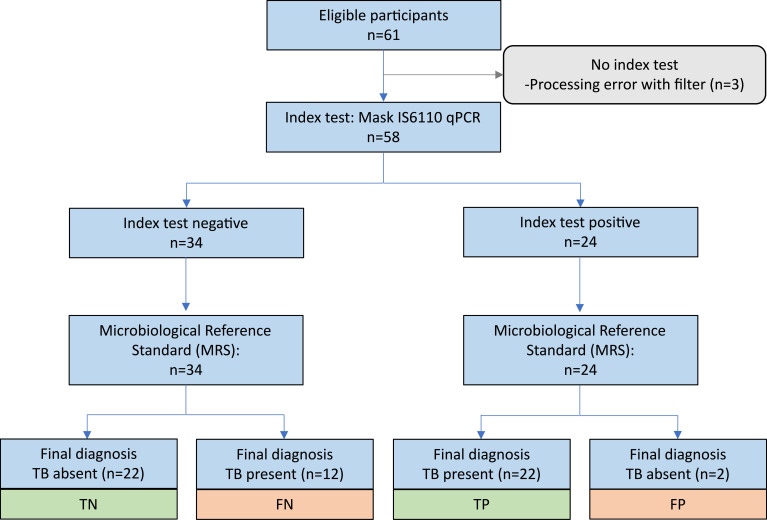
Study flow diagram.

Of the 61 enrolled participants, 3 (4.9%) were excluded from the main analysis because of filter processing errors during collection by study staff, leaving 58 participants for the main analysis (Fig. 3). The participants primarily consisted of young adults, with an equal representation of both sexes. All participants exhibited symptoms suggestive of TB, with 43% (25/58) having an HIV infection, and 29% (17/58) having a history of prior TB. There was a good agreement between the MRS and SXRS, with 59% (34/58) and 54% (31/57) of participants with confirmed TB, respectively (Table 1).

**TABLE 1:** Demographic and clinical characteristics^*a*^

Characteristic	*N* = 58
Age	41 (32, 46)
Sex at birth	
Female	31 (53%)
Male	27 (47%)
Positive WHO tuberculosis symptom screen	58 (100%)
Prior TB	
Yes	17 (29%)
No	38 (66%)
Missing	3 (5%)
People living with human immunodeficiency virus (HIV)	
HIV positive	25 (43%)
HIV negative	29 (50%)
Missing	4 (7%)
Microbiologic reference standard (MRS)	
TB positive	34 (59%)
TB negative	24(41%)
Sputum Xpert Ultra reference standard (SXRS)	
Positive	31 (53%)
High	8 (14%)
Medium	9 (16%)
Low	11 (19%)
Very Low	3 (5%)
Negative	26 (46%)
Indeterminate	1 (1%)
Trace	1 (1%)

^*a*^ Data are presented as median (interquartile range) or n (%).

#### Diagnostic accuracy of AveloMask qPCR testing

**Fig 4:**
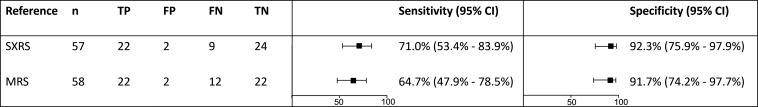
Diagnostic accuracy of AveloMask qPCR against the sputum Xpert Ultra reference standard (SXRS) and microbiological reference standard (MRS).

**Fig 5:**
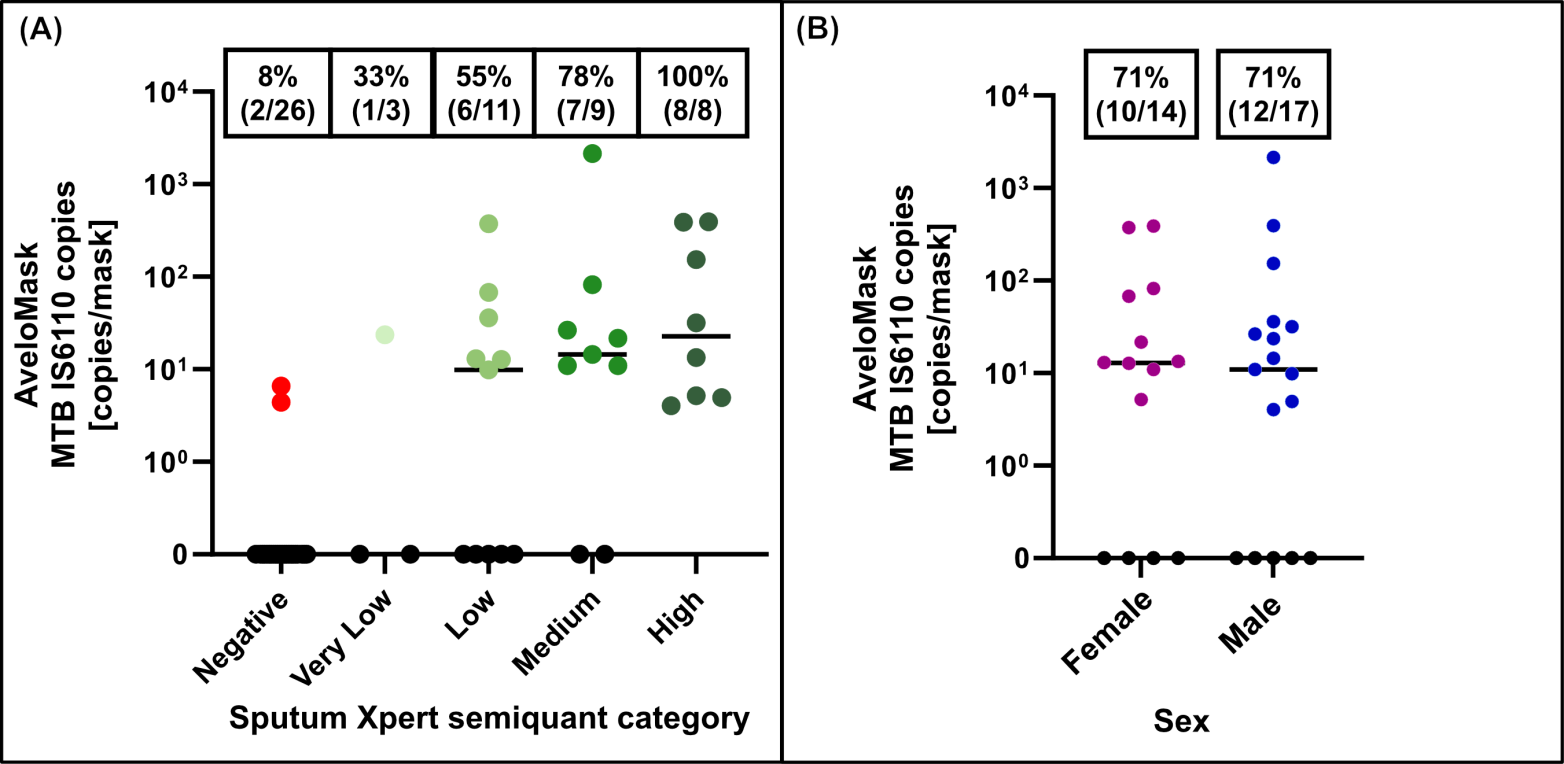
Mean number of *Mycobacterium tuberculosis IS6110* copies per AveloMask sample per participant by (**A**) Xpert Ultra semiquantitative grade and (**B**) sex.

Among the 58 participants, concordance between the MRS and mask qPCR was 75.9% (95% CI: 63.5%–85.0%). Using the MRS, mask qPCR sensitivity was 64.7% (95% CI: 47.9%–78.5%), and specificity was 91.7% (95% CI: 74.2%–97.7%). When SXRS was used as the reference, mask qPCR sensitivity was 71.0% (95% CI: 53.4%–83.9%), and specificity was 92.3% (95% CI: 75.9%–97.9%) (Fig. 4). No signs of TB were found when the medical records of the two false-positive participants were reviewed (Table S2). In both cases, positivity was observed in only one of four qPCR wells, with late cycle threshold (Ct) values (>38.5), suggesting very low positivity (Fig. 5A). Negative extraction controls included in all qPCR runs remained negative, indicating minimal risk of cross-contamination. To further investigate, a subset of DNA extracts—including those from the two qPCR-positive but clinically negative participants—were re-tested using a BLINK digital PCR (dPCR) assay. All true-positive samples were confirmed positive by dPCR, while all true-negative samples and the two initial false positives remained negative. Melt curve analysis further confirmed the presence of the correct *IS6110* amplicon in all dPCR-positive samples (Table S3).

#### Quantification of MTB copy numbers from mask samples

Detectable levels of MTB *IS6110* copies were found in 71% (22/31) of mask samples collected from participants with SXRS-positive results. The *IS6110* copy numbers spanned a 3-log range (4–2,147 copies, mean: 175 copies), and the quantities detected by qPCR were comparable to those measured by digital PCR, which enables absolute quantification (Table S3). *IS6110* copies in mask samples showed a non-significant trend toward higher values in samples corresponding to higher sputum Xpert Ultra semiquantitative categories. Sensitivity increased with higher sputum Xpert Ultra semiquantitative categories, reaching 33%, 55%, 76%, and 100% for very low, low, medium, and high categories, respectively (Fig. 5A). Mask positivity was higher among participants with high bacterial load, as indicated by shorter MGIT culture time to positivity (TTP): 100% (9/9) for samples with TTP ≤10 days compared with 36% (4/11) for samples with TTP >10 days. No significant differences in copy numbers or sensitivity were observed between sexes (Fig. 5B). When using the mean *IS6110* copy numbers from mask qPCR for ROC curve analysis, sensitivity was 61.3% when optimized for 100% specificity (Fig. S3).

To assess DNA recovery, 19 filter inlays from TB-positive participants were reprocessed. There were statistically significant higher *IS6110* copy numbers in the re-extracted samples (mean 62 vs 113 copies; *P* = 0.04), suggesting up to 65% of the DNA is missed in the initial extraction. All initially positive samples remained positive, and three of five of the initially false-negative samples were positive upon re-extraction (Fig. S4).

### User feedback

User feedback from all 61 participants indicated that the mask was generally well tolerated. Of 61 participants, 20% rated the breath sampling as “very easy,” 34% as “somewhat easy,” 36% as “okay,” 10% as “somewhat difficult,” and none as “very difficult” (Fig. S5). These findings suggest that the masks are user-friendly and feasible for use in primary care outpatient settings and among individuals presenting with symptoms of TB.

## DISCUSSION

In this study of 61 individuals presenting with symptoms of TB, breath sampling with the novel AveloMask kit for 45 min followed by qPCR detection achieved a sensitivity of 71%, indicating effective capture of exhaled MTB. This sensitivity aligns closely with other face-mask sampling studies: Kodama et al. (21) reported a sensitivity of 76% using 2-h mask sampling coupled with a similar extraction method and loop-mediated isothermal amplification (LAMP), Williams et al. (20) achieved an 86% sensitivity using qPCR following a 1-h sampling period, and Shaikh et al. (23) reported 75% sensitivity for a 10 min talk-cough-breathe process in children with PCR detection. However, unlike these previous methods, which require aseptic handling and filter manipulation with forceps, the AveloMask simplifies sample collection by enabling direct transfer of the filter inlay into an inactivating and nucleic acid-preserving buffer at the point of collection. Owing to its ease of use, the AveloMask kit demonstrated a successful collection rate of 95% (58/61) when administered by minimally trained community health workers in primary care outpatient settings.

Mask sampling was well tolerated by participants, making it an attractive alternative to sputum, which up to 18% of individuals are unable to produce and which is frequently of suboptimal quality (3–5). The high specimen availability for mask samples may offset its lower sensitivity, potentially diagnosing more individuals overall and achieving a higher diagnostic yield compared to molecular tests that require high-quality sputum specimens (7, 31). Mask sampling should further be explored for the identification of asymptomatic individuals with subclinical TB that are estimated to be responsible for 68% of global transmission (32). Since breath and cough aerosols are the primary modes of TB transmission, breath sampling may also help identify highly infectious individuals, who can be triaged for intervention strategies. Indeed, we observed higher mask qPCR positivity in individuals with high bacterial load sputum samples, suggesting mask qPCR could be used as a measure for infectiousness.

Other mask sampling use cases worth researching include self-sampling, active case finding, and treatment monitoring—including in the context of vaccine and drug development. In a study by Fennelly and colleagues (33), there was a rapid decrease in cough aerosol bacterial load within the first 3 weeks of effective treatment. Non-invasive breath sampling may also provide access to the lung microbiome (34) and improve the diagnosis of lower respiratory tract infections (35) —which similarly suffer from limited availability of representative lower airway samples. Mask sampling may further serve as a valuable tool to study respiratory transmission of diseases (36, 37) and for pandemic preparedness as masks offer the dual benefit of protecting others from transmission while enabling diagnostic sampling from the wearer. This approach could be extended to other airborne pathogens, such as influenza, SARS-CoV-2, respiratory syncytial virus (RSV), emerging zoonotic viruses, or bacteria.

Mask sampling offers practical advantages: (i) passive breath collection during waiting periods can reduce patient visit and staff time, (ii) the mask’s design may improve healthcare worker safety compared to sputum or swab collection, and (iii) collection methods are designed to be done at point-of-care. Widespread adoption, however, depends on compatibility with commercial molecular platforms. Currently, *IS6110* copy numbers in mask samples are low, requiring manual, column-based extraction. Incomplete DNA recovery suggests that actual bacillary loads are underestimated, highlighting the need to improve elution, extraction, and lysis, particularly due to MTB’s tough cell wall. Encouragingly, high sensitivity has been reported using commercial LAMP assays, indicating that improved performance with a commercial molecular assay is feasible (21). Further studies using optimized protocols are essential, in particular for Xpert Ultra, as sensitivity on mask samples from other studies was insufficient (13 to 48%) (23, 38).

As a proof-of-concept study, our aim was to demonstrate the technical feasibility and diagnostic potential of breath-based sampling with AveloMask, forming the basis for more targeted, comparative studies in relevant populations. Although AveloMask sensitivity was lower than that of sputum Xpert Ultra, it approached the sensitivities reported in oral swab studies (39–43), supporting the need for head-to-head comparisons of non-sputum-based diagnostics, particularly in populations unable to expectorate and in individuals with subclinical TB. Notably, our, as well as most sputum and tongue swab studies only enrolled sputum-producing participants, and diagnostic yield studies explicitly including non-sputum producers are now needed (7).

This study has several strengths. The AveloMask kit was carefully designed, produced, and analytically evaluated to demonstrate the capture efficiency of its novel fiber filter. It evaluates a non-invasive, breath-based sampling method that broadens TB diagnostics beyond sputum. Sample collection was conducted in real-world settings, and user feedback confirmed feasibility in relevant populations with relevant users. Blinded qPCR testing, standardized protocols for sample handling and analysis, and the inclusion of process controls and standard curves ensured the reliability and reproducibility of results. Diagnostic accuracy was evaluated using two reference standards, including a composite MRS. The study population also included both people living with and without HIV, enhancing the relevance of findings.

While this study shows promising results supporting mask sampling as a non-sputum diagnostic alternative, it has several limitations. As a small proof-of-concept study, generalizability is limited and requires validation in larger studies, which are ongoing. Potential inclusion bias may have arisen from the case-control design and the restriction to sputum-producing participants. Future studies should follow the intention-to-diagnose principle and include those unable to expectorate to better assess diagnostic yield (7, 44). As this study enrolled ambulatory participants with TB symptoms, further evaluation in more severely ill patients is needed; tolerability is currently being assessed in an ongoing study among hospitalized pneumonia patients. Usability should be formally assessed in future studies, including user preferences across relevant subgroups, including age, sex, HIV status, and disease severity relative to other sample types. The two apparent false positives were resolved using dPCR, highlighting the challenges of interpreting low DNA loads and underscoring that unspecific amplification, procedural, or environmental contamination cannot be fully excluded. Often, samples showed late Ct values (>38.5), near the assay’s detection limit. Future studies should include strategies to interpret borderline results, such as replicate-based thresholds or confirmatory testing including melt-curve analysis. qPCR-based *IS6110* copy number estimates were similar when confirmed by dPCR but should still be interpreted with caution due to variability in extraction efficiency and the stochastic nature of qPCR at low DNA concentrations. Current work is looking at optimizing the extraction protocol, which could further improve DNA recovery and thus sensitivity.

### Conclusion

The AveloMask breath aerosol sampling kit has shown promising diagnostic accuracy and feasibility in primary care settings, making it a valuable non-invasive diagnostic option for pulmonary TB. Given that at least one in five individuals struggles to produce sputum, breath-based sampling could significantly increase diagnostic yield, particularly among populations currently underserved by conventional diagnostics. Further optimization of DNA extraction methods and integration with commercial molecular testing platforms could enhance sensitivity and broaden clinical utility, ultimately contributing to earlier TB diagnosis and improved infection control.

## Data Availability

All data generated or analysed during this study are included in this article, in the supplemental material, and supplemental data spreadsheet.

## SUPPLEMENTAL MATERIAL

Supplemental movie jcm.00546-25-s0001.mp4 AveloMask Kit instructions. The movie shows the steps to collect and process an AveloMask sample

Supplemental data jcm.00546-25-s0002.csv Participant data.

Supplemental material jcm.00546-25-s0003.docx Supplemental methods, figures, and tables.

ASM does not own the copyrights to Supplemental Material that may be linked to, or accessed through, an article. The authors have granted ASM a non-exclusive, world-wide license to publish the Supplemental Material files. Please contact the corresponding author directly for reuse.
